# Controllable Assemblies of Au NPs/P5A for Enhanced Catalytic Reduction of 4-Nitrophenol

**DOI:** 10.3390/polym16152104

**Published:** 2024-07-24

**Authors:** Zhaona Liu, Bing Li, Huacheng Zhang

**Affiliations:** 1 Department of Pharmacy, Medical School, Xi’an Peihua University, Xi’an 710125, China; zhaonaliu@peihua.edu.cn; 2 School of Chemical Engineering and Technology, Xi’an Jiaotong University, Xi’an 710049, China; 3 Key Laboratory of Advanced Energy Materials Chemistry (Ministry of Education), Nankai University, Tianjin 300071, China

**Keywords:** Pillar[5]arene, Au NPs, 4-Nitrophenol, assembly, catalytic reduction

## Abstract

Efficient catalytic reduction of 4-nitrophenol (4-NP) is one focus of industry and practical engineering, because 4-NP is one of the most important sources of pollution of the ecological environment and human health. Here, coassembled hybrid composites of pillar[5]arene (P5A) and gold nanoparticles (Au NPs) were successfully developed by a one-step synthetic method as a type of water-insoluble catalyst for the reduction of 4-NP. The geometric and topological structures, as well as physiochemical properties of Au NPs/P5A composite catalyst, were fully characterized and analyzed through various tests such as transmission electron microscopy (TEM), X-ray diffraction (XRD), and Fourier-transform infrared spectroscopy (FTIR), indicating that Au NPs were well dispersed on the surface of the two-dimensional film of assembled P5A. The influence factors of the catalytic reduction of 4-NP were further investigated and discussed, confirming that the content of Au NPs and the concentration of 4-NP were very significant during the catalysis. The catalytic reaction was carried out at the catalyst concentration of 100 mg·L^−1^ and an initial 4-NP concentration of 90 mg·L^−1^ under 30 °C. The calculated reaction rate constant was 0.3959 min^−1^ and the reduction rate of 4-NP was more than 95% in 20 min. In addition, the as-prepared catalyst can maintain a high catalytic efficiency after five cycles. Thus, the easily recyclable composite catalyst with poor aqueous solution can exhibit prospective application to the treatment of 4-NP in water.

## 1. Introduction

Pollutants from industrial production are very harmful to the ecological environment and human health. As one of those pollutants, 4-nitrophenol (4-NP) is highly toxic and difficult to biodegrade, causing serious damage to the central nervous system, liver, and kidney, as well as the blood of living things [1,2]. Viewed from organic reactions and fine chemicals, 4-NP can be reduced to 4-aminophenol (4-AP), which is an important raw material used during industrial production and widely used in pharmaceuticals and dyes [3]. However, the reaction kinetics of such a transformation are very slow under natural conditions. Thus, it is of great interest to fabricate a suitable catalyst for efficiently achieving the reduction of 4-NP into 4-AP. Currently, an enormous number of metal-based catalyst materials with different forms have been developed [4,5,6]. And due to the high surface energy, metallic nanoparticles (NPs) often exhibit high catalytic activity, but suffer from the easy agglomeration during such a reaction, greatly limiting further practical applications [7]. Thus, it is necessary to find a suitable carrier to regulate and reduce the agglomeration of such metallic NPs [8].

As a new generation of macrocycles, pillararene has received extensive attention worldwide in the past decade [9,10], and its rigid molecular skeleton and electron-rich cavity award such macrocycles a unique role in application to both academic research and practical engineering, such as drug delivery systems, adsorption and separation, electrics and sensors, and catalysis [11,12]. Furthermore, due to its easy synthesis and functionalization, pillararene performs as an interesting building block in the construction of functional self-assembled composites and hybrid architectures in diverse scales and dimensions with various impressive morphologies and geometries [13,14,15]. Interestingly, it is always observed that various pillararene moieties are located on the surface of diverse metallic NPs for different applications. But by considering the difference in sizes of pillararene and metallic NPs, it is strange that bigger macrocycles occur on the surface of spatially limited metallic NPs to promote the transportation of potentially produced electrons. Thus, it is of great curiosity to apply pillararene for fabrication of complicated “larger” self-assemblies, which have advanced hierarchical structures to “hold” and stabilize smaller-sized metallic NPs to combine organic/inorganic contents for further environmental applications such as the catalytic treatment of pollutants.

Here, we designed and synthesized a heterogeneous catalyst composed of both P5A and Au NPs by one-step method, where pillararene initially performs as a useful tool in regulating the dispersion of smaller-sized Au NPs. Interestingly, such hybrid composites not only exhibit surprising morphological structures but also excellent catalytic performances in the reduction of 4-NP. Furthermore, Au NPs/P5A hybrid catalysts not only possess high catalytic activity after multiple catalytic cycles, but also accomplish good stability and recyclicity.

## 2. Experimental

### 2.1. Materials

Unless otherwise stated, all reagents were purchased from Shanghai McLean Chemical Reagent Co., Ltd. (Shanghai, China), without further purification.

### 2.2. Preparation of P5A

First, 10 g of 1,4-dimethyloxybenzene, 6.525 g of paraformaldehyde, and 230 mL of 1,2-dichloroethane were employed. The reaction was stirred at 25 °C for 30 min, and then 9 mL of boron trifluoride ether was then added as the catalyst. After 3 h of reaction at 25 °C, 200 mL of methanol was added to terminate the reaction. The product was separated and purified by rotary evaporation and filtration to afford the white solid powder of P5A.

### 2.3. Preparation of Au NPs/P5A

First, 20 mg of P5A was weighed, and 100 mL of dichloromethane was used as the solvent. The P5A was completely dissolved in the solvent by stirring at 25 °C for 10 min. Then, 3 mL of HAuCl_4_ solution with different concentrations was added to the above solution and stirred at 25 °C for 3 h to load HAuCl_4_ into the assembly of P5A. Subsequently, the temperature was increased to 45 °C, then 3 mL of sodium citrate solution of the corresponding concentration was added, stirred for 5 min, and then heated to 90 °C. In this process, HAuCl_4_ was reduced in situ to Au NPs. Finally, the Au NPs/P5A nanocomposite was obtained by repeated washing and drying. According to the different content of Au NPs, the samples were named as follows: 1 Au NPs/P5A, 2 Au NPs/P5A, 4 Au NPs/P5A, 6 Au NPs/P5A, and 8 Au NPs/P5A.

### 2.4. Catalytic Reduction Experiment

We weighed 10 mg sodium borohydride as a reducing agent, dissolved 3 mL in a 4-NP solution with a concentration of 90 mg·L^−1^, and added a certain amount of composites. Then, the absorbance (400 nm) and spectral curve (250–480 nm) of the reduced solution were measured by a UV–Vis spectrophotometer. The concentration after reduction can be determined by the standard curve of 4-NP, and the reduction rate is used to represent the catalytic performance of the catalyst.

### 2.5. Techniques of Characterizations

The Fourier-transform infrared spectroscopy (FTIR) was measured in the range of 4000 to 400 cm^−1^ using a ThermoFisher FTIR spectrophotometer (Nicolet iS50) (Shanghai, China) with KBr tableting method. The powder X-ray diffraction (XRD) pattern was collected by a Shimadzu LabX XRD-6100 X-ray diffractometer (Tokyo, Japan). Transmission electron microscopy (TEM) studies were performed on an FEI Talos F200x instrument (Shanghai, China). The measurement of absorbance was carried out on a TU-1901 UV–Vis spectrophotometer (Tokyo, Japan), with quantitative measurements at 400 nm or scanning spectra at 250–480 nm.

## 3. Results and Discussion

### 3.1. Characterization of the Au NPs/P5A Composite

The Au NPs/P5A composite was prepared via one-step method by reducing HAuCl_4_ into Au NPs in the presence of P5A in mixed solutions of dichloromethane and water under programmable heating, as described in the experimental section.

**Figure 1:**
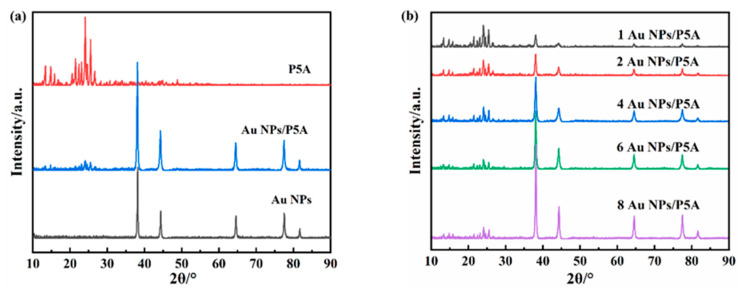
XRD patterns of Au NPs/P5A composites by comparing to pristine P5A and Au NPs (**a**), as well as by changing the amount of Au NPs in those composites (**b**).

To detect the structural pattern of this obtained Au NPs/P5A composite, X-ray diffraction (XRD) detection was carried out to compare the pristine P5A, Au NPs, and the hybrid composite, as shown in Figure 1. The diffraction peaks appeared at 2θ = 38.2°, 44.4°, 64.4°, and 77.8°, corresponding to the (111), (200), (220), and (311) crystal planes of Au NPs, respectively, which proved that HAuCl_4_ was reduced successfully during the formation of Au NPs (Figure 1a). In addition, the characteristic peaks of P5A and Au NPs simultaneously appeared in the XRD pattern of the hybrid composite of Au NPs/P5A, demonstrating the coassembly behaviors of Au NPs and P5A. Furthermore, Figure 1b shows the same characteristic peaks in the XRD patterns of Au NPs/P5A composites but with different contents of Au NPs, showing that the intensity of characteristic peaks of Au NPs/P5A catalysts was strengthened with the increase in Au NPs. Thus, sharp diffraction peaks by XRD clearly indicate that the as-prepared Au NPs/P5A composites possess desired crystallinity.

**Figure 2:**
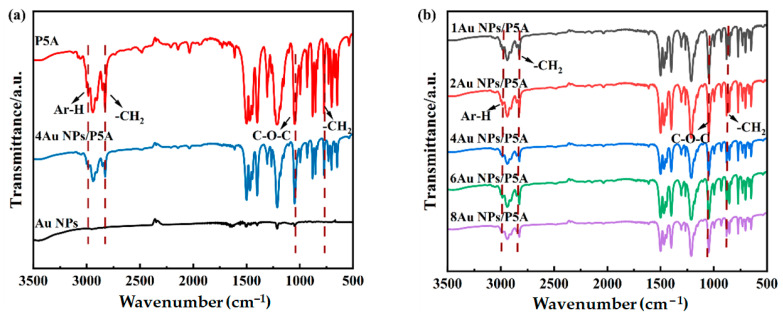
Comparison of FTIR spectra of P5A, Au NPs, and Au NPs/P5A (**a**), as well as spectra of hybrid composites with diverse amounts of Au NPs (**b**).

To further evaluate the effect of the presence of Au NPs in the hybrid composite on the chemical structure of P5A, FTIR was employed. As shown in the spectrum of P5A in Figure 2a, the characteristic absorption peaks at 3047 cm^−1^, 2930 cm^−1^, 750 cm^−1^, and 1047 cm^−1^ correspond to C-H on the benzene moieties, the tensile vibration mode of -CH_2_, the bending vibration mode of -CH_2_, and the absorption peak of C-O-C in P5A, respectively. The FTIR spectra hardly reflect the structural information of Au NPs, but a weak fluctuation at 1390 cm^−1^ can be observed in the Au NPs spectrum (Figure 2a), which is the characteristic absorption peak of the carboxyl group, due to the trace amount of sodium citrate employed in the reduction of Au NPs. In addition, those characteristic absorption peaks show up in the spectrum of Au NPs/P5A without any new peaks, indicating that the chemical structure of P5A was not destroyed in the presence of Au NPs in the hybrid composite. Furthermore, Figure 2b shows the compared FTIR spectra of Au NPs/P5A composites with different amounts of Au NPs, revealing similar characteristic peaks to those in the spectrum of pristine P5A.

**Figure 3:**
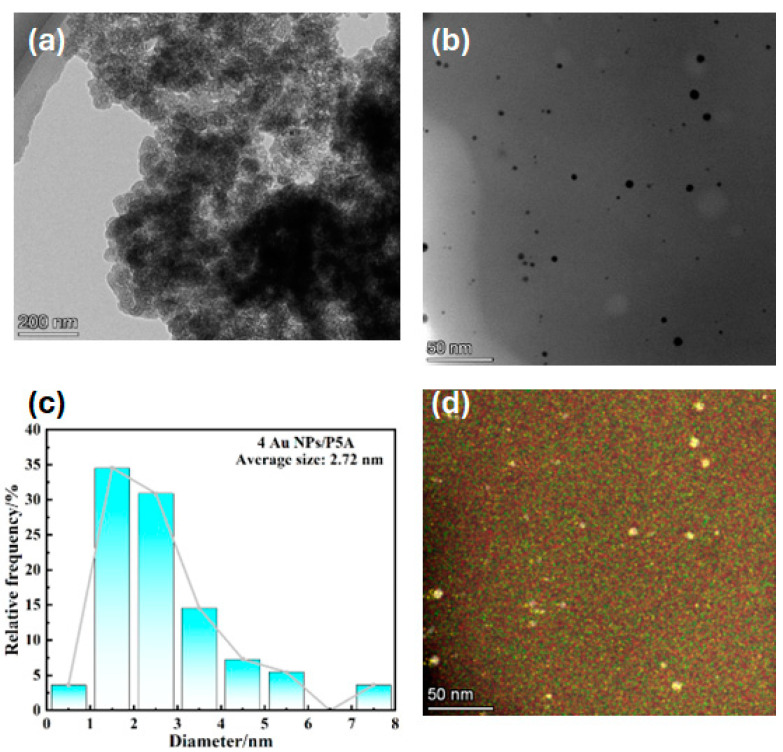
Comparison of TEM images of Au NPs in the absence (**a**) and presence of P5A (**b**), the particle size distribution of Au NPs/P5A as statistically calculated by TEM observations (**c**), and TEM mapping technology employed for distinguishing diverse key elements in hybrid composites (**d**).

Morphologies of Au NPs/P5A hybrid composites were further observed by using transmission electron microscopy (TEM), as shown in Figure 3. The Au NPs exhibit two different morphologies, such as agglomeration and good dispersion in the absence and presence of P5A (Figure 3a,b), respectively. Surprisingly, the film-like pillararene-based assemblies were initially recorded in the figures, showing the smooth, widespread, two-dimensional characteristic. The Au NPs were embedded on the surface, providing a possible spatially limited position for further holding valuable “targets”. Furthermore, the narrow particle size distribution of nanosized Au NPs/P5A, as statistically calculated by TEM observation in Figure 3b, is shown in Figure 3c, and matches well in both figures. In addition, TEM mapping technology (Figure 3d) also indicates the distribution of significant elements such as C, O, and Au on the hybrid composite of Au NPs/P5A, proving that Au NPs were well dispersed on the surface of the organic film assembled by P5A.

### 3.2. Catalytic Performance of Au NPs/P5A on Reducing 4-NP

**Figure 4:**
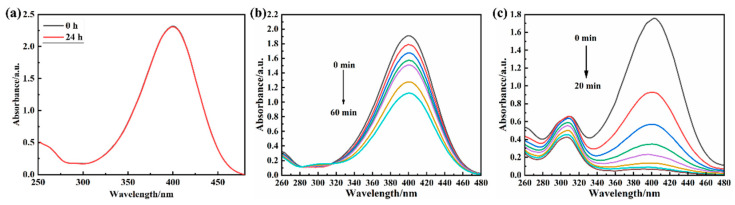
UV–Vis detection of catalytic reduction of 4-NP in the presence of P5A (**a**), Au NPs (**b**), and Au NPs/P5A (**c**).

The performance of different catalysts, i.e., Au, Au NPs/P5A, and P5A, were initially tested regarding the catalytic reduction efficiency of reducing 4-NP, as shown in Figure 4. The control experiment using P5A as the named “catalyst” had no catalytic effect on the decomposition of 4-NP after 24 h as shown in Figure 4a with no differences between red and black lines. In addition, another control experiment using pristine Au NPs as the catalyst did show a bit of a catalytic effect, but with poor catalytic performance. The reduction rate of 4-NP in that case was only 50% after 60 min (Figure 4b). Finally, the Au NPs/P5A composite as the catalyst exhibited a higher catalytic effect, achieving a reduction rate of more than 90% within 20 min (Figure 4c). Thus, the Au NPs/P5A composite does exhibit better catalytic performance than the pristine Au NPs. This might be caused by the agglomeration of Au NPs being significantly reduced with the assistance of P5A in the hybrid composite.

**Figure 5:**
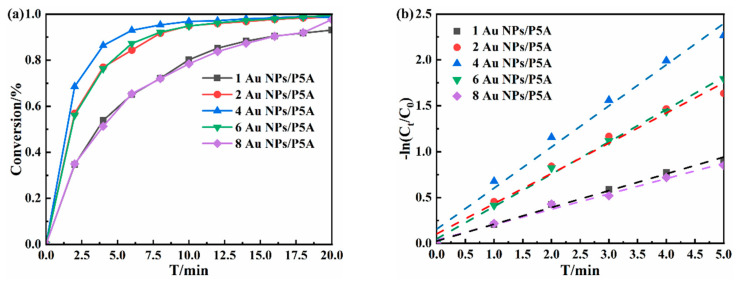
Catalysts with different loadings in reduction of 4-NP: the change in 4-NP conversion rate with time (**a**), and the fitting curve according to the reaction rate (**b**).

Furthermore, the catalytic performance of such composite with different amounts of loaded Au NPs for the reduction of 4-NP was thoroughly tested, as shown in Figure 5 and Table S1. It was found that the catalytic rate of 1 Au NPs/P5A was low, and the catalytic reduction conversion rate of 4-NP was also low within 20 min, with the conversion rate at 90%, due to the lower content of Au NPs. With the increase in loaded Au NPs, the conversion rate of 4-NP simultaneously increased gradually, due to the accordingly increased catalytic active sites and the subsequently improved catalytic efficiency. Particularly, the sample of 4 Au NPs/P5A had the best catalytic effect, while with the further increase in Au NPs, the conversion rate of 4-NP showed a downward trend, due to the possibility of agglomeration of particles in the limited space on the surface of the P5A film, leading to the poor dispersion of particles as well as stacked active sites.

**Figure 6:**
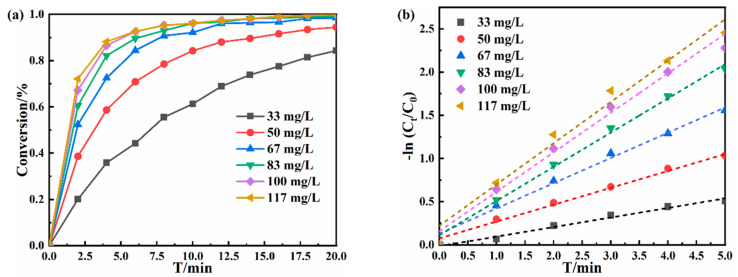
Catalytic conversion diagram (**a**) and catalytic rate fitting diagram (**b**) of different catalyst dosage.

The concentration of such hybrid catalyst is important for affecting the catalytic performance as well as the expansion of scale production and application. The catalyst concentration was adjusted from 33 mg·L^−1^ to 117 mg·L^−1^, and the obtained effect on catalytic performance is shown in Figure 6. With the increase in catalyst dosage, the catalytic conversion of 4-NP showed a trend of increasing first and then stabilizing within 20 min. The catalytic rate gradually increased, but the rate of increase gradually slowed down, as shown in Figure 6b. Usually, the slope of the fitting curve reflects the catalytic rate, and, in accordance, the larger the slope, the greater the reaction rate will be. The specific fitting k value is shown in Table S2. In the case with the low catalyst dosage, there are fewer active sites, showing the low catalytic rate, and the catalytic conversion of all substrates cannot be completed within 20 min. Then, the increase in catalyst dosage with more Au NPs leads to the increase in catalytic active sites accordingly. Thus, the catalytic rate is increased, and the conversion rate is reached in 20 min. When the concentration of the catalyst exceeds 100 mg·L^−1^, the catalytic rate increases slowly instead. This might be caused by the concentration of the catalyst reaching the optimal value; a further increase in the amount of catalyst will make the active sites overlap. Thus, the increase in catalytic rate does not increase linearly with the increase in the concentration of the catalyst, but leads to the trend of slower growth. By considering both the cost of catalysts and the influence of catalytic efficiency, 100 mg·L^−1^ was chosen as the optimal dosage in the subsequent experiments.

**Figure 7:**
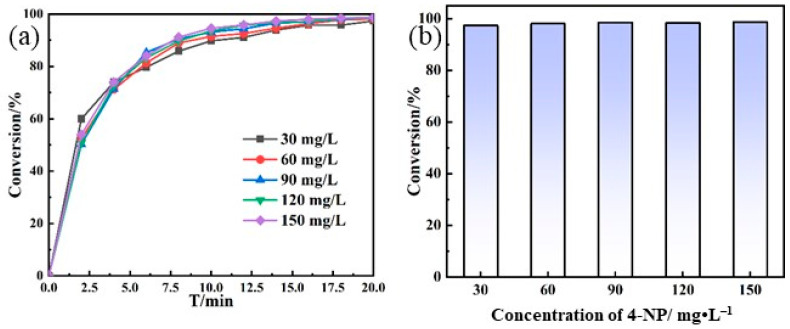
The catalytic conversion rate diagram of different initial concentration of 4-NP: (**a**) the change in time and (**b**) the conversion rate reached in 20 min (**b**).

The effect of the initial concentration of the substrate, 4-NP, on the catalytic performances such as the conversion rate was then evaluated, as shown in Figure 7. The results showed that there were no significant differences in the conversion rate from either the change in time (Figure 7a) or the conversion rate reached in 20 min (Figure 7b). Thus, the prepared catalyst is applicable in a relatively wide concentration range and maintains high catalytic activity. By considering that the industrial wastewater does not contain the high concentration of 4-NP, the concentration of 90 mg·L^−1^ for initial 4-NP is used in subsequent experiments.

**Figure 8:**
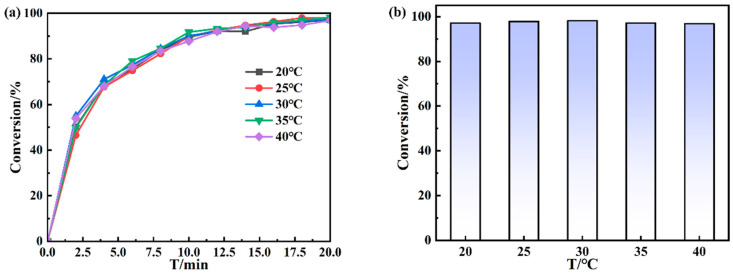
Catalytic conversion diagram at different temperatures: (**a**) change in conversion rate with time (**a**) and the conversion rate reached in 20 min (**b**).

Additionally, the effect of temperature on the catalytic performance is shown in Figure 8. The conversion rate of the catalytic process was determined by the temperature of the catalytic system in the range from 20 °C to 40 °C. The results, including both the change in conversion rate with time (Figure 8a) and the conversion rate reached in 20 min (Figure 8b), showed that the temperature had no significant effects on the catalytic performance. Thus, the prepared catalyst can be applied to the general scale of temperatures, reserving stable catalytic activity and reasonable stability, which is key for the further production and application of such catalyst.

**Figure 9:**
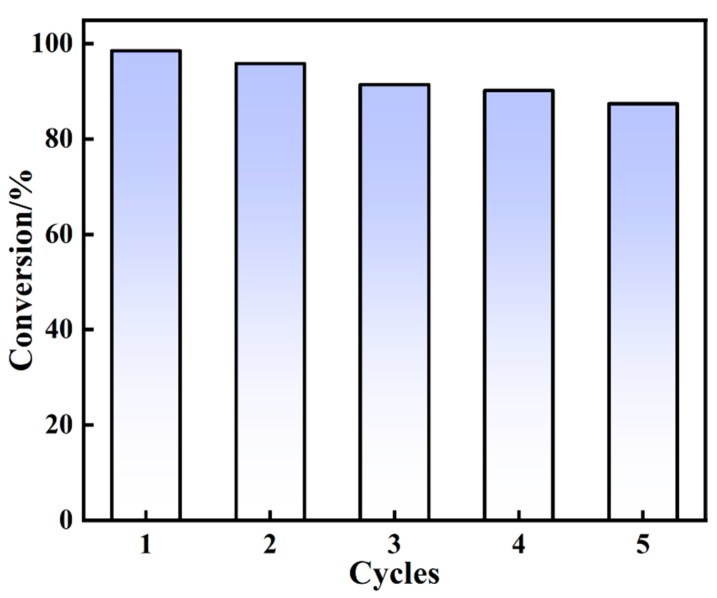
Regeneration performance of catalyst for 4-NP reduction reaction.

The regeneration performance of the catalyst affects the cost of production in practical engineering and industrial applications, which is an important indicator to evaluate the quality of the catalyst. First, 90 mg·L^−1^ of initial 4-NP was selected for the reaction at room temperature. Then, the composite sample of 4 Au NPs/P5A at the concentration of 67 mg·L^−1^ was used as the catalyst, and NaBH_4_ was used as the reducing agent. After the catalytic reaction was completed, the used 4 Au NPs/P5A catalyst was further washed and dried, and the repeated catalyzation was then carried out five times, as shown in Figure 9. As the recyclization number increased, the catalytic conversion rate of 4-NP by the hybrid composite gradually decreased from 98.57% to 87.45%. The catalytic efficiency does have the trend of decreasing, but the composite after recycling still maintains a reasonable catalytic activity, indicating that the Au NPs/P5A can be repeatedly used for the catalytic reduction of 4-NP.

## 4. Catalytic Mechanism

The reduction of 4-NP by the hybrid composite, Au NPs/P5A, includes similar general steps to other heterogenous catalysts such as adsorption, catalysis, and desorption. For example, the 4-NP is absorbed on the surface of Au NPs/P5A composites and reduced by NaBH_4_, and 4-AP is also desorbed from the hybrid composite. Thus, effects on accelerating these three steps can promote the reduction reaction rate accordingly.

The reason why the Au NPs/P5A composite can effectively improve the reduction rate of 4-NP is as follows. First, the concept of host–guest inclusion in supramolecular chemistry can assist in explaining the catalytic mechanism of Au NPs/P5A. For example, the hydrophobic and electron-deficient cavity of P5A with benzene subunits can promote the adsorption of the targeted hydrophobic molecule, 4-NP, with the electron-withdrawing nitro moiety by the hybrid composite in water via a series of supramolecular interactions such as hydrophobic, π–π stacking, and donor–acceptor interactions. Thus, when the catalytic reaction is complete, the -NH_2_ moiety on the product, 4-AP, is not only the electron-donating group, but also the hydrophilic subunit, which can greatly weaken its interactions with the P5A in the hybrid composite and finally accelerate its desorption after the catalysis.

Additionally, the synergistic effect in the construction of the obtained hybrid composite also plays a unique role in promoting such a reduction reaction. For example, the P5A assembled into the two-dimensional film, as shown by TEM observation, and further performed as the carrier to effectively disperse Au NPs. The good dispersion of Au NPs indicates the specialized “dot matrix”, as well as provides the enlarged specific surface area and more electron holes for the transportation of electrons. Additionally, the anion of BH_4_^−^ from the reducing agent NaBH_4_ can be adsorbed on the surface of the hybrid composite to serve in the reduction of 4-NP into 4-AP.

## 5. Conclusions

The Au NPs/P5A coassembled composite was successfully synthesized by a one-step solvothermal method and employed to catalyze the reduction of 4-NP into 4-AP. The effects of the ratio between Au NPs and P5A on the morphology and physicochemical properties of Au NPs/P5A nanocomposites were thoroughly studied. Surprisingly, the film-like pillararene-based assemblies were initially observed by TEM, showing the smooth, two-dimensional surface for dispersing Au NPs. It also provided the possibility for attracting valuable targets such as 4-NP in the fabricated spatially limited “dot matrix”. The catalytic experiment was then carried out to investigate the effect of diverse conditions on the catalytic reduction performance. By utilizing the optimal ratio of Au NPs and P5A, the effects of amounts and concentration of Au NPs, temperature, and initial concentration of 4-NP, as well as other factors, on the catalytic reduction reaction were systematically analyzed. Finally, the reaction mechanism of Au NPs/P5A in the catalytic reduction of 4-NP was discussed. Macrocyclic molecules such as P5A assisting in dispersion of Au NPs, a series of supramolecular interactions for promoting the process of adsorption/desorption towards the substrate/product, and the synergistic effect of organic–inorganic composites are supposed to play important roles in such homogenous catalysis.

## Data Availability

The original contributions presented in the study are included in the article/Supplementary Materials, further inquiries can be directed to the corresponding author/s.

## Supplementary Materials

The following supporting information can be downloaded at: https://www.mdpi.com/article/10.3390/polym16152104/s1, Table S1 The catalytic rate k value of the catalyst with different amount of Au NPs. Table S2 The catalytic rate k value of different catalyst dosage.
