# Supplementary material for: Controllable Assemblies of Au NPs/P5A for Enhanced Catalytic Reduction of 4-Nitrophenol
Source: Polymers (Basel). 2024 Jul 24;16(15):2104. doi: 10.3390/polym16152104 (PMC11314206; doi:10.3390/polym16152104)
Supplement: Supplementary file 1 [file polymers-16-02104-s001.zip › polymers-3090121-supplementary.pdf]

# Controllable Assemblies of Au NPs/P5A for Enhanced Catalytic Reduction of 4-Nitrophenol

**Zhaona Liu [1], Bing Li [2] and Huacheng Zhang [2,3,*]**

[1] Department of Pharmacy, Medical School, Xi'an Peihua University, Xi'an 710125, China; zhaonaliu@peihua.edu.cn
[2] School of Chemical Engineering and Technology, Xi'an Jiaotong University, Xi'an 710049, China
[3] Key Laboratory of Advanced Energy Materials Chemistry (Ministry of Education), Nankai University, Tianjin 300071, China
* Correspondence: zhanghuacheng@xjtu.edu.cn

Table S1 The catalytic rate k value of the catalyst with different amount of Au NPs.

| Catalyst | Mass/mg·$L^{-1}$ | K/$min^{-1}$ |
|---|---|---|
| 1 Au NPs/P5A | 100 | 0.1829 |
| 2 Au NPs/P5A | 100 | 0.3294 |
| 4 Au NPs/P5A | 100 | 0.4480 |
| 6 Au NPs/P5A | 100 | 0.3524 |
| 8 Au NPs/P5A | 100 | 0.1671 |

Table S2 The catalytic rate k value of different catalyst dosage.

| Catalyst | Mass/mg·$L^{-1}$ | K/$min^{-1}$ |
|---|---|---|
| 4 Au NPs/P5A | 33 | 0.1106 |
| | 50 | 0.1951 |
| | 67 | 0.2920 |
| | 83 | 0.3959 |
| | 100 | 0.4570 |
| | 117 | 0.4778 |
